# *Leishmania* development in sand flies: parasite-vector interactions overview

**DOI:** 10.1186/1756-3305-5-276

**Published:** 2012-12-03

**Authors:** Anna Dostálová, Petr Volf

**Affiliations:** 1 Department of Parasitology, Faculty of Science, Charles University in Prague, Vinicna 7, 128 44, Praha 2, Czech Republic

**Keywords:** *Phlebotomus*, *Lutzomyia*, Kinetoplastida, Proteolytic enzymes, Peritrophic matrix, Chitinase, Innate immunity

## Abstract

Leishmaniases are vector-borne parasitic diseases with 0.9 – 1.4 million new human cases each year worldwide. In the vectorial part of the life-cycle, *Leishmania* development is confined to the digestive tract. During the first few days after blood feeding, natural barriers to *Leishmania* development include secreted proteolytic enzymes, the peritrophic matrix surrounding the ingested blood meal and sand fly immune reactions. As the blood digestion proceeds, parasites need to bind to the midgut epithelium to avoid being excreted with the blood remnant. This binding is strictly stage-dependent as it is a property of nectomonad and leptomonad forms only. While the attachment in specific vectors (*P. papatasi*, *P. duboscqi* and *P. sergenti*) involves lipophosphoglycan (LPG), this *Leishmania* molecule is not required for parasite attachment in other sand fly species experimentally permissive for various *Leishmania*. During late-stage infections, large numbers of parasites accumulate in the anterior midgut and produce filamentous proteophosphoglycan creating a gel-like plug physically obstructing the gut. The parasites attached to the stomodeal valve cause damage to the chitin lining and epithelial cells of the valve, interfering with its function and facilitating reflux of parasites from the midgut. Transformation to metacyclic stages highly infective for the vertebrate host is the other prerequisite for effective transmission. Here, we review the current state of knowledge of molecular interactions occurring in all these distinct phases of parasite colonization of the sand fly gut, highlighting recent discoveries in the field.

## Review

### Introduction

The genus *Leishmania* (Kinetoplastida: Trypanosomatidae) are protozoan parasites causing a spectrum of diseases called leishmaniases, in their vertebrate hosts, including humans. There are about ten *Leishmania* species of significant importance for public health. Symptoms of leishmaniases can range from mild self-healing cutaneous lesions to fatal visceral cases. The lack of a human vaccine, increasing resistance to the currently used drugs and their serious side effects urge the need for research of leishmaniasis. In particular, studies focusing not only on the parasite itself, but also its interactions with its hosts and vectors are needed. For example, it is not known yet if drug-resistant *Leishmania* strains develop well in sand flies and retain their resistance after the passage through the vector.

The parasite has a digenetic life-cycle alternating between a mammalian host and insect vectors, phlebotomine sand flies (Diptera: Psychodidae, subfamily Phlebotominae). These are small (usually 1.5 – 2 mm body length) insects that are principally found in tropical and subtropical regions. Females of two sand fly genera, *Phlebotomus* and *Lutzomyia*, are of medical importance as the only proven vectors of *Leishmania* species pathogenic for humans [1].

Outside the vertebrate host, the *Leishmania* life cycle is confined to the digestive tract of sand flies. The precise location differs between subgenera *Leishmania* and *Viannia*. The New World subgenus *Viannia*, e.g. *Leishmania braziliensis*, enter the hindgut before migrating forward into the midgut and are therefore called peripylarian parasites. However, most *Leishmania* species (subgenus *Leishmania*) are suprapylarian parasites as their development is restricted to the midgut [2]. As suprapylarian parasites have been used in most studies on parasite–vector interactions, most of the mechanisms discussed below apply to this subgenus unless stated otherwise. Development in the vector is initiated when female sand flies ingest blood containing macrophages infected with amastigotes, small (3–5 μm), immotile and rounded form of the parasite. The change in conditions moving from the mammalian host to the sand fly midgut (such as decrease in temperature and increase in pH) triggers morphological transformation and development of the parasite in the vector. The amastigotes transform into procyclic promastigotes - weakly motile forms with a short flagellum beating at the anterior end of the cell. These are the first replicative forms that proliferate in the early blood meal and are separated from the midgut by a type I peritrophic matrix. Around 48–72 hours later, parasites begin to slow their replication and differentiate into strongly motile long nectomonad promastigotes [3]. These escape from the peritrophic matrix-encased blood meal into the midgut lumen. They move towards the anterior midgut and later develop into short nectomonad promastigotes [4], also called leptomonads [3], which enter another proliferative cycle [5-7]. Detachment, forward migration and colonization of the stomodeal valve are essential for effective transmission. Ultimately, *Leishmania* transform into infective metacyclic stages [8], which are delivered to the skin of the vertebrate host during the next blood feeding.

Molecular background of some of these interactions has been partially characterized while others remain yet to be uncovered. While the genome sequences of several *Leishmania* species have been published [9] and molecular studies abound, molecular data on sand flies are limited. Genome sequencing projects of two phlebotomine species, *Phlebotomus papatasi* and *Lutzomyia longipalpis*, are in progress [10,11] but a final assembly and annotation have not been published yet for either species. Besides studies characterizing sand fly population structure and phylogeography [12-16] an analysis of expressed sequence tags (ESTs) from the whole *L. longipalpis* sand fly and salivary gland transcriptomes of several sand fly species have been published [17-20]. With regard to *Leishmania* development in the midgut, particularly midgut-specific transcriptomic analyses of *L. longipalpis*, *P. papatasi* and *Phlebotomus perniciosus*[21-24] have brought important insights into the repertoire of molecules expressed in the midgut.

Here we review the current state of knowledge of the complex interactions of the *Leishmania* parasite with its insect vectors, summarizing natural barriers to *Leishmania* development in various phases of infection. Several recent studies have brought important insights into the molecular bases of challenges posed by the vector digestive tract environment and the adaptations developed by the *Leishmania* parasite and we highlight these recent discoveries in the field.

Studies elucidating parasite-vector interactions have become the basis for novel approaches to reduce transmission of several insect-borne diseases. For example, they led to the use of novel vector-based transmission-blocking vaccines (TBVs) [25]. TBVs aim at preventing the transmission of pathogens by targeting molecule(s) expressed on the surface of pathogens during their developmental phase within the insect vector or by targeting molecules expressed by the vectors in salivary glands [26,27] or midgut [28]. This strategy has been used successfully in identifying promising vaccine candidates for malaria control [29] and has a great potential in the research of leishmaniases.

### Early phase survival

Natural barriers to *Leishmania* development within the sand fly gut during the first few days after blood feeding include secreted proteolytic enzymes, the peritrophic matrix surrounding the ingested blood meal and most likely sand fly immune reactions.

#### Proteolytic enzymes

In the midgut of unfed sand flies there is little baseline protease activity. It is the ingestion of blood meal that induces secretion of digestive enzymes. Significant levels of protease activity are detected 6h post blood meal (PBM) and peak levels are reached 18-48h PBM depending on the sand fly species. The pH optimum of the general endoprotease activity is in the alkaline range (7.5-9.5). Based on the results of assays using specific inhibitors, it can be attributed to serine proteases, namely trypsin- and chymotrypsin-like enzymes [30,31].

More recently, sequencing of ESTs has identified numerous transcripts coding for putative digestive enzymes in *P. papatasi, P. perniciosus* and *L. longipalpis*, the most abundant of them indeed being trypsins and chymotrypsins [17,21-24]. Temporal expression profiles of putative trypsins (3 to 4 different molecules described in the midgut of each species) show that one or several trypsin transcripts are present in high abundance in sugar fed females while their quantities decrease after the intake of blood. At the same time, the expression of other putative trypsins is induced upon blood feeding [24,32,33]. Recently, Telleria *et al*. [31] have detected the expression of one of the *L. longipalpis* midgut trypsins (LlTryp1 [GenBank: ABM26904.1]) also at the protein level. The observed protein expression profile corresponded to the transcript levels detected previously, confirming LlTryp1 as a blood-feeding induced molecule.

The second most abundant digestive enzymes in the sand fly midgut are chymotrypsins. Three to six different molecules have been identified in the midgut of each species. The expression patterns of these chymotrypsins are similar to the above mentioned trypsin molecules indicating that there may be early and late classes of serine proteases in sand flies, similar to what has been observed in some mosquitoes [34,35].

Besides trypsins and chymotrypsins, transcripts for other putative digestive proteases have also been described in the sand fly midgut. These include metallocarboxypeptidases, astacin-like metalloproteases, an alanyl aminopeptidase and a novel serine protease [18]. Aminopeptidase activity has also been detected in the sand fly midgut after blood feeding, mainly associated with the midgut epithelium [30].

It has been recognized for a long time that the activity of digestive enzymes affects *Leishmania* development in sand flies. Several studies showed reduced parasite numbers and even dead or destroyed parasites in the midguts of ‘non compatible’ sand fly species in the early phase of infections, that is the time of the onslaught of the proteolytic activity [36-38]. Based on a pioneer study by Adler (1938) [39], one of these studies [38] also reported enhanced survival of *L. donovani* in *P. papatasi* following meals devoid of serum and showed that this was correlated with delayed timing and decreased levels of peak protease activities. Moreover, other studies revealed that even in ‘compatible’ parasite-vector combinations, up to 50% of the initial amastigote parasite inoculum is killed within the first day after blood feeding [3,40].

Several publications have identified the digestive enzymes as one of the culprits of these early parasite losses using various methods to suppress midgut proteolytic activity. The addition of soybean trypsin inhibitor to the blood meal promoted the early survival of *L. donovani* in *P. papatasi*[41]. The addition of the inhibitor also enhances survival of ‘compatible’ parasites in sand flies, in which the formation of the peritrophic matrix has been blocked by chitinase [40,42]. Under normal conditions the peritrophic matrix is thought to act as a partial barrier limiting the exposure of the parasites to the digestive enzymes in the earliest phases of infection (for more details see section ‘Peritrophic matrix proteins and chitinases’). Volf *et al*. [43] report an enhancing effect of heparin on *L. major* infections in its natural vector *P. duboscqi*. The authors attribute this effect to the fact that heparin suppressed midgut trypsin activity, while it did not affect defecation, oviposition or mortality of the sand flies. This finding also stresses the importance of using defibrinated or citrated blood instead of a heparinized one in experimental infections of sand flies trying to mimic natural conditions. Recently, Sant'Anna *et al.*[44] have demonstrated that specific knock-down of the blood feeding induced trypsin (LlTryp1) in *L. longipalpis* promotes the survival of *L. mexicana*.

In order to complete its developmental cycle, *Leishmania* must have developed mechanisms to overcome the hostile environment of the blood fed midgut. Numerous studies provide evidence that the parasite manipulates the levels and timing of protease activity in the midgut. Schlein and Romano [45] and Dillon and Lane [46] demonstrated the ability of *L. major* to suppress or delay the peak of trypsin and aminopeptidase activity in the midgut. Similar observations have recently been made in *L. longipalpis*. Both *L. mexicana*[44] and *L. infantum*[31] infections led to a decreased trypsin activity in the midgut. However, it should be noted that survival of *L. major* in *P. papatasi* and *P. duboscqi* has also been observed in the absence of any significant inhibition of or delay in peak protease activities during infection [40,47].

Transcriptomic studies have revealed that the presence of *L. major* and *L. infantum* in the midgut of their natural vectors can affect the abundance of several digestive enzyme transcripts after blood feeding. Both over- and underrepresentation of some digestive enzyme transcripts in infected flies have been reported [21,22,31]. Recent quantitative analysis of *P. perniciosus* midgut trypsin expression has shown a slight decrease in the abundance of the main blood feeding-induced trypsin molecule in *L. infantum* infected sand flies [24]. These observations suggest the ability of the parasites to modulate the expression of the vector`s proteases. More detailed studies at both transcriptomic and biochemical level are needed to show how this modulation affects the resulting proteolytic activities in the midgut and elucidate the mechanism of this modulation.

Another way the parasites might affect the gut proteolytic activity has recently been suggested. Serine protease inhibitors (ISPs) were found in *L. major* despite a lack of potential target enzymes in the genome of the parasite [48]. The ISPs have inhibitory effects against vertebrate macrophage serine proteases, such as neutrophil elastase and one of them (ISP2, [GeneDB: LmjF.15.0510]) has been shown to enhance parasite survival in murine macrophages [49]. The ISPs also inhibit trypsin-like activity of sand fly midguts *in-vitro*[50]. The possibility of ISPs having an effect on insect midgut proteases *in-viv*o is currently under investigation in our laboratory.

Perhaps most importantly, *Leishmania* parasites also possess mechanisms that increase their resistance to proteolytic attack without inhibiting the overall proteolytic activity in the midgut. Pimenta *et al.*[40] exposed *L. major* to lysates of blood-fed *P. papatasi* midguts *in vitro*. Fresh tissue amastigotes and fully differentiated promastigotes were relatively resistant, whereas parasites within early stage amastigote-to-promastigote transition (2–8 h) became highly susceptible to killing. The authors interpret their observation as resistance of promastigotes as well as fully transformed promastigotes to the midgut proteolytic activity. The identification of molecules that might play a role in defending the parasite against proteolytic damage has focused on a family of glycoconjugates, the phosphoglycans (PG), that incorpororate the common structure of repeating [Gal-Man-PO4] units. These molecules are either attached to the cell surface through glycosylphosphatidylinositol lipid anchors, including the lipophosphoglycan (LPG) and the proteophosphoglycan (PPG), or they are secreted as protein-containing phosphoglycans, including the secreted proteophosphoglycan (sPPG) and a secreted acid phosphatase [51]. The results of Secundino *et al*. [47] identify *L. major* surface PPG as the likely key molecule conferring resistance of fully developed procyclic promastigotes to the activity of digestive enzymes showing that (i) a parasite line lacking surface PGs is more susceptible to killing than the wild type when exposed to blood-fed midgut lystes *in-vitro*, (ii) this effect is in great part reversed by the addition of purified PPG or (iii) by the addition of trypsin inhibitor. Protection conferred by PPG was not associated with inhibition of enzyme activities, but with cell surface acquisition of this molecule. The observed detrimental effect can likely be attributed to specific activities of sand-fly midgut trypsin-like enzymes as the same parasite line lacking surface PGs proliferates well when exposed to high concentrations of bovine trypsin [52]. Alternatively, the effect may result from a combined action of midgut trypsins in concert with other, as yet unidentified, factors present in the midgut lysate.

Taken together, sand fly midgut proteolytic enzymes are one of the critical factors affecting *Leishmania* development in the vector and represent attractive targets for vector-based transmission blocking strategy. Examples have been set in the research of malaria, where promising vaccine candidates have been identified being able to block *Plasmodium* ookinete development in the mosquito midgut [53]. *Anopheles gambiae* midgut carboxypeptidase B has been shown to be up-regulated by *Plasmodium* infection and antibodies against one of these enzymes blocked parasite development in the mosquito midgut [54]. Similarly, antibodies targeting an *A. gambiae* membrane aminopeptidase disrupted the development of *Plasmodium falciparum* and *P. berghei* ookinetes [55].

#### Peritrophic matrix proteins and chitinases

The peritrophic matrix (PM) is an extracellular chitin-containing envelope, which in most insects separates the gut lumen from the midgut epithelium. It is composed of chitin, proteins, and glycoproteins [56]. In nematoceran Diptera, including sand flies, females produce a type 1 PM, which is secreted by the midgut epithelium in direct response to the distension of the midgut caused by blood feeding [57,58]. The structure of the sand fly peritrophic matrix is complex and rearranges during the course of blood digestion. Within several hours PBM a thin PM composed mainly of chitin fibrils covers the whole surface of the blood bolus. At later stages (12h-2 days PBM depending on the sand fly species) the PM gets thicker and matures. Proteins and glycoproteins are incorporated in its structure and heme incrustations also appear. Sequentially (2-3 days PBM), the PM structure appears wrinkled and then starts to break down [59-61].

At the molecular level, several proteins putatively participating on the PM formation and breakdown in sand flies have been identified. The main class includes peritrophins, proteins contatining chitin binding domains (CBDs). Two types of putative peritrophin molecules have been identified in the midgut transcriptomes of *P. papatasi, P. perniciosus* and *L. longipalpis*: multiple-CBD peritrophins and single-CBD proteins [21,22,24]. Some of them contain predicted -N and/or -O type glycosylation sites including mucin-type domains. The putative peritrophins with multiple CBDs are likely to have a role in cross-linking the chitin fibrils of the peritrophic matrix. Single-CBD peritrophins may have roles in capping the ends of chitin fibrils or sequestering free chitinous molecules within the midgut lumen. In addition to chitin binding, mosquito proteins with CBDs have also been described to bind heme and have a role in its sequestration during blood digestion [62]. Glycosylation of the PM proteins can be of great importance for the PM structure and function. Heavily glycosylated proteins, such as peritrophins containing mucin domains, can influence the selectiveness of the PM pores and account for water retention within the PM. Glycosylation can also influence susceptibility to degradation by temporally secreted digestive proteases: aglycosylated PM proteins are likely to be more prone to proteolytic clevage resulting in changes in the PM thickness and structure [56].

Interestingly, different peritrophin expression patterns were described in sand fly species differing in their vector competence (see section ‘Establishment of infection: attachment of the parasites to the midgut epithelium’ for vector classification). In the midgut of *P. papatasi*, a peritrophin transcript was highly abundant before blood feeding and thereafter down-regulated [21]. In contrast, in two broadly permissive species, *P. perniciosus* and *L. longipalpis*, most of the sequences originated from the library of midguts after blood feeding [22,24]. Comparative transcriptomic studies have shown modulation of peritrophin transcript abundance by the presence of *Leishmania* parasites. *Phlebotomus papatasi* infected with *L. majo*r down regulated a multi-domain peritrophin (PpPer1, [GenBank: ABV44705]), whereas *L. longipalpis* infected with *L. infantum* up regulated the orthologous peritrophin (LuloPer1, [GenBank: ABV60306]) [21,22]. The significance of this finding remains unclear. Besides peritrophins, non-chitin binding proteins have recently been identified in the PM of *Anopheles gambiae*[63] and their homologs were also found in the sand fly midgut [24].

The major role in PM breakdown has been attributed to chitinases. Chitinolytic activity in the sand fly midgut is induced after the intake of blood and peaks around 48h PBM. Ramalho-Ortigao *et al.*[64] described a functional, blood-induced chitinolytic system, in the midgut of *P. papatasi* and named the identified enzyme PpChit1 [GenBank: AAV49322]. It was produced as a recombinant protein and antibodies against this protein inhibit the midgut chitinolytic activity *in vitro*. The authors presume that PpChit1 is involved in the maturation and degradation of *P. papatasi* PM (and a similar role is assumed for its orthologs, LlChit1 [GenBank: AAN71763] and PperChit [GenBank: EZ933285] in *L. longipalpis* and *P. perniciosus*, respectively) [24,64,65].

Several studies suggest a dual role for the sand fly PM regarding *Leishmania* development: it protects the parasites against proteolytic attack at the beginning of digestion yet becomes a barrier to parasite escape when mature. Pimenta *et al*. [40] observed that blocking the PM formation in *P. papatasi* midgut by addition of chitinase in the blood meal leads to a sharp increase in the number of *L. major* parasites killed within a few hours PBM. Early parasite mortality was reversed by the addition of soybean trypsin inhibitor. The authors conclude that the PM creates a barrier to the rapid diffusion of digestive enzymes, and limits the exposure of parasites to these enzymes during the time when they are especially vulnerable to proteolytic damage [40]. On the other hand, at later phases the PM appears to act as a barrier to the parasite development. Long nectomonads must escape from the endoperitrophic space to prevent being passed together with remnants of the digested blood meal. Walters *et al*. [66] reported entrapment of *L. panamensis* in the endoperitrophic space of *P. papatasi*. The failure of the parasite to escape from the PM in an inappropriate vector resulted in their expulsion from the midgut. Pimenta *et al*. [40] further showed that addition of allosamidin, a chitinase inhibitor, to infective blood meal led to thickening of the PM and entrapment of *L. major* within the peritrophic space thus preventing further development of the parasite in its natural vector *P. papatasi*. Recent data also indicate that an anterior PM plug, the part of PM secreted by thoracic midgut and located at the junction between the anterior and posterior midgut acts as a barrier to *Leishmania* migration towards the stomodeal valve [60].

Schlein *et al.*[67] first proposed that *Leishmania* escape from the PM is accomplished by a parasite chitinase. They described *L. major* escaping at the anterior end of the PM in *P. papatasi*. Further work supported their hypothesis by showing that *L. mexicana* chitinase-over expressing strain had an accelerated escape from the PM in *L. longipalpis*[42]. However, as described by Schlein and Jacobson [68], *Leishmania* chitinase is inhibited by the presence of hemoglobin. In a recent study, Sadlova and Volf [60] suggest that *L. major* chitinase does not have an important role in the disintegration of the PM in *P. duboscqi*. The detailed histological and electron-microscope study did not reveal any signs of PM lysis caused by *Leishmania* and showed that the PM opens similarly in uninfected and infected females. *Leishmania major* parasites were shown to have escaped from the posterior end of PM opened at the end of blood meal digestion (lysed presumably by the activity of vector chitinase). Importantly, Coutinho-Abreu *et al.*[69] report that knock-down of *P. papatasi* chitinase, PpChit1, by the means of RNAi led to a significant reduction in the number of *L. major* present in the midgut 120h PBM. It can be concluded that the parasites taking advantage of the sand fly chitinolytic activity within the midgut is the main mechanism for their escape. Taken together, the PM plays important roles in the parasite development and proteins involved in its formation, maturation and disintegration provide a promising target for transmission blocking vaccines.

#### Proteins and peptides involved in innate immunity

Innate immune response plays an important role in the control of bacterial and parasitic infections in the midgut of bloodsucking insects [70,71]. So far, very few studies have addressed this question in sand flies. Defensins, cationic antibacterial peptides, have been described in the fat body and the midgut. In *P. duboscqi*, defensin [Swiss-Prot: P83404] was induced by both bacteria and *Leishmania* infection and the recombinant peptide showed a significant anti-parasitic activity against *L. major in vitro*[72]. Transcripts coding for several other putative components of the innate immune response have been detected in the sand fly midgut, such as pattern recognition proteins, a glycin-rich protein and serpins. Moreover, homologs of antioxidant enzymes, molecules that are known to regulate midgut epithelial immunity and impact the outcome of bacterial and parasitic infections in mosquitoes, have also been found [21-24,73,74]. A recent study has demonstrated that in *L. longipalpis*, depletion of Caspar [GenBank: AM093416], a putative negative regulator of immune deficiency signaling pathway, by the means of RNAi prior to blood feeding, leads to a significant reduction of populations of both *L. mexicana* and *L. infantum*. This result suggests that activation of the immune response can control *Leishmania* development in the vector [75]. Moreover, feeding reactive oxygen species (namely H_2_O_2_) or silencing catalase [GenBank: ABV60342], an anti-oxidant enzyme, both showed detrimental effects on *L. mexicana* development in the midgut [76]. The role of individual recognition and effectors molecules and precise orchestration of both midgut and systemic immune homeostasis await further investigation.

### Establishment of infection: attachment of the parasites to the midgut epithelium

As the blood digestion proceeds, parasites need to bind to the midgut epithelium to avoid being excreted with the blood remnant. Following the escape from the endoperitrophic space, the parasites attach to the midgut, inserting their flagella between the epithelial microvilli. There is no obvious ultrastructural modification of the flagellum associated with midgut binding. It remains unclear whether the involvement of the flagellum is essential *per se*, or merely a reflection of the fact that being at the anterior end the flagellum will contact the epithelium first and fits between the microvilli [77]. By anchoring themselves to the midgut the parasites help to prevent their expulsion from the gut during defecation, and it has been postulated that this binding is the main determinant of parasite-vector specificity [78,79]. Our recent *in-vitro* binding study showed that *Leishmania* gut binding is strictly stage-dependent, is a property of those forms found in the middle phase of development (nectomonad and leptomonad forms), but is absent in the early blood meal and final stages (procyclic and metacyclic forms) [80].

Based upon experimental tests of their ability to support development of wide or limited range of *Leishmania* species, sand flies have been classified as specific (also called restrictive by some authors) or permissive vectors [81]. Most sand fly species tested to date support development of multiple *Leishmania* species and are thus called ‘permissive vectors’. In contrast, there appears to be a close evolutionary fit between *P. papatasi* and *P. duboscqi* with *L. major* and *P. sergenti* with *L. tropica*, as other *Leishmania* species survive poorly in these sand fly hosts [6,52]. The mechanism of parasite attachment has been most intensively studied in the specific vector *P. papatasi* infected with *L. major*.

#### Phlebotomus papatasi

The attachment of *Leishmania major* in its specific vector *P. papatasi* is the most studied parasite-sand fly interaction so far. The role of parasite surface lipophosphoglycan (LPG) has been demonstrated by a series of studies. LPG is an abundant glycolipid that covers the entire surface, including the flagellum, of all *Leishmania* promastigote stages. The basic LPG structure is highly conserved in all *Leishmania* species. It consists of a glycosyl-phosphatidyl-inositol lipid anchor attached through a hexasaccharide core to a polymer of 10–30 PG repeating units terminated by a small neutral oligosaccharide cap [82]. The PG repeating units are often modified by strain-, species-, and stage-specific side-chain sugar residues. Purified *L. major* LPG was shown to bind to dissected *P. papatasi* midguts [83] and inhibit the binding of *L. major* promastigotes to the midgut *in vitro*[84]. More recent studies using LPG-deficient parasites confirmed the crucial role of LPG in the attachment of *L. major* in the midgut. These mutants lack the LPG1 gene which encodes a galactofuranosyltransferase required for synthesis of the LPG glycan core, rendering such cells specifically deficient in LPG. The ability to persist in the midgut of *P. papatasi* following blood meal excretion was completely lost in these parasites and this defect was correlated with their inability to bind to midgut epithelial cells *in vitro*[79]. A similar observation was recently made with these mutants in another specific vector, *P. duboscqi*[52].

In order to produce a transmissible infection in the sand fly, the parasites need to be able to detach from the midgut epithelium and produce free-swimming metacyclic forms. In *L. major* - *P. papatasi* combination the attachment is achieved by stage-specific modifications in the LPG structure. Parasite binding is mediated by modified phosphoglycan repeats bearing side chain galactosyl residues [84]. During metacyclogenesis, the original LPG is replaced by metacyclic form LPG, which has increased numbers of PG repeats and side-chain galactose residues masked by the addition of terminal arabinose [85]. Thus modified metacyclic form LPG does no longer bind to the *P. papatasi* midgut [84].

Based on the finding of the role of sugar residues in the attachment hypothesis, it was postulated that lectins or lectin-like molecules serve as receptors for parasite binding in the midgut. Lectin-like activities have indeed been described in the sand fly midgut [86-88]. Sequencing of a *P. papatasi* midgut cDNA library led to the discovery of a galectin molecule (PpGalec, [GenBank: AAT11557.1]) that was proved to serve as a receptor for *L. major* LPG [89]. PpGalec is a 35kDa galectin containing two non-identical carbohydrate recognition domains. It is continuously expressed throughout the development of larval and pupal stages, but is strongly up-regulated in adult females. It appears to be restricted to the midgut, despite lacking a signal peptide, it is expressed on the luminal surface of *P. papatasi* midgut epithelial cells. The role of PpGalec in *L. major* binding was proven by several experiments. PpGalec produced as a recombinant protein bound specifically to *L. major* promastigotes bearing side-chain galactose residues on their LPG *in vitro*. Antibodies directed against this protein blocked *L. major* binding to midguts *in vitro* and severely impaired the parasites` development *in-vivo* when fed to *P. papatasi* in the infectious blood meal [89]. Interestingly, the binding of recombinant PpGalec to promastigotes was not only species-specific (recognizing neither *L. tropica* nor *L. donovani*), but also strain-restricted. Significant binding was only observed with the Friedlin V1 strain of *L. major* (Israeli isolate), sympatric to the *P. papatasi* used in the study (a colony originating from the Jordan Valley). A West African Seidman strain (SD) of *L. major*, with LPG virtually devoid of galactose side-chains as well the LV39 strain (Central Asia isolate), with long poly-galactose side chains failed to bind recombinant PpGalec (of the Jordanian flies). These observations are in accordance with the earlier findings that the LV39 grow poorly in the Cyprus as well Jordanian colonies of *P. papatasi*[90,91] and the SD strain does not survive in *P. papatasi* at all [92]. Using *L. major* lines mutated in galactosytransferases, Dobson *et al*. [93] have recently characterized an LPG side-chain galactosylation pattern optimal for survival in *P. papatasi* originating from the Jordan Valley. The key element is the presence of mostly mono-galactosylated PG repeats. However, the study also reveals that the optimal galactosylation pattern, while being a prerequisite, is not on its own sufficient for the binding to occur. Coating *L. donovani* with the optimally galactosylated LPG did not confer its survival in the midgut of *P. papatasi*, parasites being lost most likely due to the failure to bind to the epithelia. The authors suggest the existence of an additional, as yet uncharacterized *L. major*-specific ligand that is required for successful binding and survival in the midgut. Whether this additional ligand binding could also explain the fact that attachment usually occurs via the flagellum, whereas LPG is found over the whole surface of promastigotes, remains to be elucidated. Thus, in spite of being the best characterized *Leishmania*-sand fly interaction, the binding of *L. major* in its natural vector *P. papatas*i is not yet fully understood.

Furthermore, in the above mentioned study using an *in-vitro* binding assay [80] we observed that both *L. braziliensis* and *L. tropica* were able to bind to the midgut in significant numbers when competing with the natural parasite *L. major*. Neither of these species is able to complete their development in *P. papatasi in vivo*. These results show that although gut binding may be necessary for parasite establishment, the specificity of such *in vitro* binding alone is insufficient to explain overall vector specificity.

#### Other sand fly species

While the role of LPG in the attachment of *L. major* in *P. papatasi* and *P. duboscqi* has been unambiguously proved, the necessity of LPG on the parasite surface and the nature of receptors for parasite binding in the midgut are still in question in other sand fly species. The structure of LPG side-chains is highly species- and in some cases strain-specific. Similarly to *L. major*, the structure of LPG is different in metacyclic parasites than in other forms in other *Leishmania* species. For example, in an Indian strain of *L. donovani* the PG repeats are modified with glucose and this modification is down-regulated during metacyclogenesis, along with increasing the length of the PG backbone [94]. In contrast, in a Sudanese strain of this species there are no side chains modifications at all [95].

After the identification of *L. major* LPG as the parasite ligand for binding in *P. papatasi* midgut, a number of studies have been carried out implicating a similar role for LPG in other *Leishmania* species. Pimenta *et al*. [78] observed binding of purified LPG from several *Leishmania* species (*L. donovani*, *L. major*, *L. amazonensis*) to the midguts of *P. argentipes in vitro*, corresponding to the ability of the parasites to survive in *P. argentipes* in laboratory infections. In contrast, *P. papatasi* midguts were only stained with LPG purified from *L. major*. A similar high specificity was found for *L. tropica* in its vector *P. sergenti*; midguts were intensely stained following incubation with purified PG from *L. tropica* compared with PGs from *L. major* or *L. donovani*[96]. Soares *et al*. [97] blocked the binding of *L. infantum* to dissected midguts of its natural vector *L. longipalpis* by purified PG of this species.

Despite results implying LPG in the parasite attachment, neither receptors in the sand fly midgut nor the mechanism of parasite release in the later phase of infection have been sufficiently characterized in sand flies other than *P. papatasi*. Expression of the tandem repeat galectin (PpGalec) seems to be restricted to *P. papatasi* and *P. duboscqi*, as shown by a genomic dot blot as well as immunoblot (using antisera raised against this protein) with a variety of sand fly species [89]. No galectin sequences were found in the midgut transcriptome of a permissive vector species, *P. perniciosus*[24]. In the analysis of the *L. longipalpis* midgut-specific transcriptome, one low-abundance transcript was identified, which is homologous to a single-domain galectin [22]. Given that this sand fly species supports development of a wide range of *Leishmania* species including those whose LPG is not galactose-modified and therefore is not expected to be recognized by galectins, it is unlikely that this galectin acts as a receptor for *Leishmania* in *L. longipalpis*. No molecular data are available for other sand fly species and the nature of putative LPG receptors remains unclear.

Pimenta *et al*. [78] suggested that midguts of *P. argentipes* possess a receptor for a conserved part of LPG, accounting for the broad permissivity to various *Leishmania* species. In their later study, the authors suggest that the binding of *L. donovani* in *P. argentipes* occurs via receptors for saccharides present in the neutral LPG cap that is masked by conformational changes in the elongated PG chains in metacyclic *L. donovani*[98]. It should be noted that despite sharing some common features (all are composed of neutral hexoses), the LPG caps show remarkable interspecies differences. They vary both quantitatively and qualitatively in the content of mannose, galactose or glucose, raising uncertainty about the nature of a putative common receptor.

Importantly, we have recently observed LPG-independent development of *Leishmania* in four permissive vectors, *P. arabicus*, *P. argentipes, P. perniciosus* and *L. longipalpis*. *Leishmania major lpg1*^*-*^ line devoid of LPG survived well and developed mature infections fully comparable to wild type parasites in these sand flies [52,99]. Similarly, Rogers *et al*. [100] report that *L. mexicana lpg1*^*-*^ mutants survive and complete their development in *L. longipalpis*. These results contradict those reported by Pimenta *et al.*[78]. In their study, *L. donovani* LPG-deficient mutant line ‘R2D2’ failed to survive in *P. argentipes*. However, the R2D2 was obtained following heavy mutagenesis and selection for LPG deficiency [101], leaving the possibility that non-specific deleterious effects account for the observed phenotype. Restoration of the LPG1 gene expression to R2D2 only weakly restored survival in *P. argentipes*[90].

Myskova *et al*. [99] hypothesised that LPG is required in specific vectors, while in permissive vectors *Leishmania* bind via an LPG independent mechanism. They observed a correlation between the occurrence of N-acetyl-D-galactosamine- displaying glycoconjugates in the midgut of sand flies and their permissivity. They suggest these glycoconjugates as ligands for *Leishmania* attachment in permissive vectors and show the binding of such molecules in the midgut lysate of *P. halepensis* to *L. major* promastigotes *in vitro*. This new binding modality implies involvement of a parasite lectin-like receptor. The authors propose heparin binding proteins that had been previously described on the surface of various *Leishmania* species [102-104] as potential candidates. In agreement with this hypothesis, de Castro Cortes *et al*. have recently observed heparin binding proteins from the surface of *Leishmania (Viannia) braziliensis* promastigotes participating in the adhesion of parasites to *L. longipalpis* cell line *in vitro*[105,106]. However, the cell line can hardly mimmic highly differenciated midgut cells with microvilli.

It should be noted that the distinction of sand flies into two categories only, specific and permissive vectors, is a working concept that likely oversimplifies the real situation. It is clear that much still remains to be learned about the mechanisms of attachment on both the parasite and the vector side.

### Late-stage development

Ultimately, *Leishmania* transform into infective metacyclic stages and their delivery to the skin of the vertebrate host must be ensured for effective transmission. Metacyclics are small, rapid-swimming forms with an elongated flagellum that originate from leptomonads [5]. It has been shown that metacyclogenesis in *Leishmania* is induced *in vitro* by low pH and nutrient depletion, while reduced tetrahydrobiopterin levels may also act as a signal for parasite differentiation [77,107]. Functional endosome sorting and autophagy are required for metacyclogenesis in *Leishmania in-vitro*[108] and the genetic locus encoding HASPs and SHERP, *Leishmania*-specific proteins of unknown function, is essential for metacyclogenesis of *L. major* in *P. papatasi*[109]. Very little is known about the actual signals triggering metacyclogenesis in the sand fly midgut. Of interest, a V-ATPase has been recently described in the midgut of *L. longipalpis* that could be involved in gut acidification [110].

Occasionally, *Leishmania* metacyclics were observed in salivary glands of sand flies [111] or in urine droplets discharged by infected females during blood feeding [112]. However, it is generally accepted that there are two main mechanism of transmission of metacyclic parasites: either a limited number of metacyclics occurring in the proboscis is deposited into the skin during feeding [113] or parasites residing behind the stomodeal valve (the junction between anterior midgut and foregut) are regurgitated with a backflow of ingested blood [100]. Originally, the regurgitation was supposed to result from the mechanical block of the foregut or the stomodeal valve [114]. More recently, the damage to the chitin layer of the stomodeal valve [67] and the role of parasite proteophosphoglycan [100] were described.

During late-stage infections, large numbers of short nectomonad and metacyclic parasites accumulate in the anterior midgut. These parasites are packed in filamentous proteophosphoglycan (secreted most probably by the short nectomonad stages) creating a gel-like plug physically obstructing the gut [100,115]. Further contributing to the blockage of the gut is another form of the parasite called haptomonad forms. These leaf-like parasites are attached to cuticular lining of the stomodeal valve through an expanded flagellar tip containing hemidesmosomal structures. The ultrastructure of these hemidesmosomes has been known for many years, but it remains to be biochemically described both at the parasite and the vector side. The attached parasites cause damage to the structure of the stomodeal valve, likely interfering with its function and facilitating reflux of parasites from the midgut [116,117]. The destruction is likely due to the action of parasite secreted chitinase [42].

In a recent study, Kimblin *et al*. [118] performed quantification of *L. major* promastigotes deposited into the skin by single *Phlebotomus duboscqi* females. They observed a bimodal distribution of the numbers of transmitted parasites: most of the sand flies delivered a low infectious dose (<600 parasites), while the remainder transmitted much higher doses, corresponding also to a higher percentage of the parasites present in the midgut before blood feeding. The authors suggest that this bimodality reflects the two distinct mechanisms of transmission. Mimicking high- and low-dose transmission by intradermal needle infections, they show that the inoculum size impacts on the outcome of the infection. Large lesions developed rapidly in the ears of mice receiving the high-dose inoculum while the low dose resulted in only minor pathology but a higher parasite titer in the chronic phase [118]. Interestingly, Maia *et al*. [119] report higher parasite loads transmitted by sand fly vectors for an *L. infantum* strain with dermal tropism as compared to a viscerotropic strain of the same species. Collectively, these data suggest that the infectious dose might be one of the determining factors in the outcome of *Leishmania* infection. Other factors affecting parasite establishment in the skin of the vertebrate host and modulating the local immune response, such as sand fly saliva and proteophosphoglycan forming promastigote-secretory gel, have been reviewed [7,26,27] and do not fit in the scope of this paper.

## Conclusions

**Figure 1:**
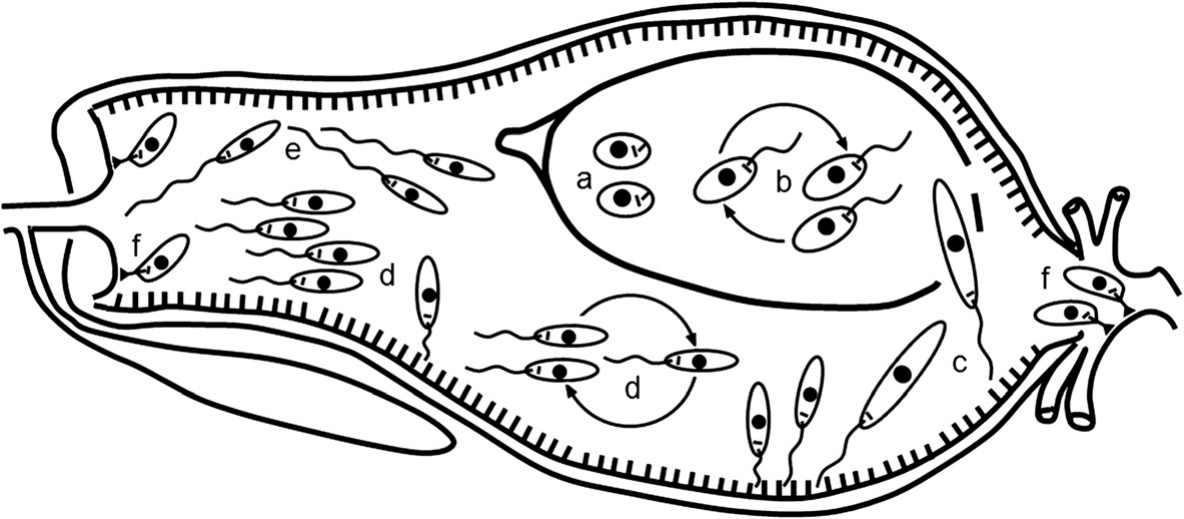
**Development of *Leishmania *in the sand fly digestive tract.** Sand fly midgut is composed of a single layered epithelium with a brush border of microvilli lining the lumen. In contrast, the foregut (including the stomodeal valve) and the hindgut (including the pyloric triangle) are lined by chitin. Amastigotes (**a**) ingested along with a bloodmeal into abdominal midgut transform into procyclic promastigotes (**b**), these replicate and transform to long nectomonads (**c**). During the bloodmeal digestion the parasites are surrounded by peritrophic matrix (PM). When the PM is broken by sand fly enzymes, long nectomonads escape through the posterior opening and attach to midgut microvilli. The next stage are replicative short nectomonads called leptomonads (**d**); these transform into infective metacyclic promastigotes (**e**) or attach to the chitin lining of the stomodeal valve as haptomonads (**f**). In the late-stage development, masses of nectomonads secreting filamentous proteophosphoglycan obstruct the thoracic midgut. This, together with destruction of the valve, facititates reflux of parasites when the fly takes a subsequent bloodmeal. In subgenera *Viannia* and *Sauroleishmania*, haptomonads attach also to chitin lining of the pylorus region.

*Leishmania* life cycle in the vector gut includes several morphological forms, some of them assumed to be non-dividing (long nectomonads and metacyclics) and some prolipherating vigorously (Figure 1). Significant advances have been made in exploring *Leishmania*-vector interactions in recent years. With accumulating, sometimes contradictory, data we start to explore how complex, and in many cases species-specific, these interactions are. Lipophosphoglycan (LPG) is involved in attachment of long and short nectomonads to midgut epithelium in specific vectors *P. papatasi* and *P. duboscqi* while in permissive sand fly vectors *Leishmania* bind via an LPG-independent mechanism. Phosphoglycans, but not LPG, are required for resistence of procyclic forms to sand fly digestive enzymes. Immune-related sand fly molecules, when activated, seem to adversely impact the development of *Leishmania* in the midgut. *Leishmania* chitinase is likely not required for escape of long nectomonads from the peritrophic matrix-encased blood meal into the midgut lumen but in late-stage infections causes the damage to the chitin lining of the stomodeal valve. This pathological change, together with obstruction of the thoracic midgut by gel-like plug composed of sPPG, facilitates parasite transmission.

Studies applying modern technologies have brought important insights into several aspects of this parasite-vector system, in some cases correcting the “old dogmas”. New approaches and midgut-specific transcriptomes of several sand fly species have provided a catalogue of molecules potentially important for the vectorial competence of sand flies and a handful of functional studies identify some of them as new targets for vector control.

## Abbreviations

TBVs: Transmission-blocking vaccines; ESTs: Expressed sequence tags; PM: Peritrophic matrix; PBM: Post blood meal; CBD: Chitin binding domain; LPG: Lipophosphoglycan; PG: Phosphoglycan; ISP: Inhibitor of serine peptidases; PPG: Proteophosphoglycan; sPPG: Secreted proteophosphoglycan.

## Competing interest

The authors declare that they have no competing interests.

## Authors’ contributions

AD wrote the initial draft. Both authors read and approved the final manuscript.
